# Age-specific effects of estrogen receptors' polymorphisms on the bone traits in healthy fertile women: the BONTURNO study

**DOI:** 10.1186/1477-7827-7-32

**Published:** 2009-04-22

**Authors:** Francesco Massart, Francesca Marini, Gerolamo Bianchi, Salvatore Minisola, Giovanni Luisetto, Antonella Pirazzoli, Sara Salvi, Dino Micheli, Laura Masi, Maria Luisa Brandi

**Affiliations:** 1 Department of Pediatrics, University of Pisa, Pisa, Italy; 2 Department of Internal Medicine, University of Florence, Florence, Italy; 3 Rheumatology Unit, Azienda Sanitaria 3, Genova, Italy; 4 Department of Clinical Sciences, II Clinica Medica, Rome, Italy; 5 Endocrine Unit, University of Padua, Padua, Italy; 6 GlaxoSmithKline-Italia, Verona, Italy

## Abstract

**Background:**

Skeletal characteristics such as height (Ht), bone mineral density (BMD) or bone turnover markers are strongly inherited. Common variants in the genes encoding for estrogen receptor alpha (ESR1) and beta (ESR2) are proposed as candidates for influencing bone phenotypes at the population level.

**Methods:**

We studied 641 healthy premenopausal women aged 20–50 years (yrs) participating into the BONTURNO study. Exclusion criteria were irregular cyclic menses, low trauma fracture, metabolic bone or chronic diseases. Serum C-telopeptide of type I collagen (CTX), osteocalcin (OC), and N-terminal propeptide of type I procollagen (P1NP) were measured in all enrolled subjects, who underwent to lumbar spine (LS), total hip (TH) and femoral neck (FN) BMD evaluation by DXA. Five hundred seventy Caucasian women were genotyped for ESR1 rs2234693 and rs9340799 and ESR2 rs4986938 polymorphisms.

**Results:**

Although no genotype differences were found in body parameters, subjects with combined ESR1 CCGG plus ESR2 AA-AG genotype were taller than those with opposite genotype (P = 0.044). Moreover, ESR1 rs2234693 genotypes correlated with family history of osteoporosis (FHO) and hip fracture (FHF) (P < 0.01), while ESR2 AA-AC genotypes were strongly associated with FHF (OR 2.387, 95% CI 1.432–3.977; P < 0.001).

When clustered by age, 20–30 yrs old subjects, having at least one ESR1 rs2234693 C allele presented lower LS- (P = 0.008) and TH-BMD (P = 0.047) than TT genotypes. In 41–50 yrs age, lower FN-BMD was associated with ESR2 AA (P = 0.0180) subjects than in those with the opposite genotype. ESR1 rs2234693 and rs9340799 and ESR2 rs4986938 polymorphisms did not correlate with age-adjusted values of OC, CTX and P1NP.

**Conclusion:**

These findings support the presence of age-specific effects of ESR1 and ESR2 polymorphisms on various skeletal traits in healthy fertile women.

## Background

Bone mass increases during the growth period and peaks by young adulthood. Although the greatest gain in bone mass takes place during the accelerated growth in adolescence, bone mineral density (BMD) continues to increase for several years (yrs) later [1].

The importance of peak bone mass as a determinant of osteoporosis and fractures later in life is supported by several studies. For instance, Hui et al. [2] estimated that peak bone mass and postmenopausal bone loss contributed equally to bone status in 70-year-old women. Hernandez et al. [3] estimated that a 10% increase in peak BMD may delay the development of osteoporosis by 13 yrs.

Low BMD is, indeed, a major determinant of osteoporotic fractures, even though environmental factors, such as dietary intake and physical activity play an important role in the BMD determination. From studies of monozygotic and dizygotic twins, inheritance was estimated to account for 60–80% of BMD in both men [4] and women [5]. Many other predictors of fragility fracture, bone turnover markers and skeletal geometry are also under genetic control. In the last two decades, an exceptionally wide range of candidate genes have been proposed as risk markers of osteoporosis outcomes [6], but our ability to predict which patients are most likely to sustain low BMD and/or osteoporotic fractures based on genetic screening is still far to be complete.

Among the analyzed candidate genes are those encoding estrogen receptor α (*ESR1*) and β (*ESR2*). In particular, single nucleotide polymorphisms [7] defined by the restriction enzymes *Pvu*II (*rs2234693, C/T*) and *Xba*I (*rs9340799, A/G*) in the *ESR1 *intron 1, and by *AluI *(*rs4986938, A/G*) in the 3'-untraslated region (3'*UTR*) of *ESR2 *exon 8 have been evaluated in more than 90 population-based studies, with inconclusive results. For their specific ethnic distribution, clinical predictability of estrogen receptor polymorphisms are strongly dependent on the analysis of homogenous populations [8].

The purpose of this study was to relate skeletal traits such as height (Ht), BMD and bone turnover markers, measured in a large cohort of healthy premenopausal Caucasian women aged 20–50 yrs, to several genotyped polymorphisms in the *ESR1 *(*rs2234693 *and *rs9340799*) and *ESR2 *(*rs4986938*) loci.

## Methods

### Subject population

The analyzed population included all enrolled participants in the Bone Turnover Range of Normality (BONTURNO) study, a multicenter, multiracial/multiethnic cohort study from young adulthood to midlife. The design of the BONTURNO has been described in detail [9]. A total of 641 subjects were enrolled from 20 different centers uniformly distributed across Italy. Each center was asked to recruit four to six healthy individuals for each age range: 20–24, 25–29, 30–34, 34–39, 40–44, and 45–49. A prerequisite for the screening was the presence of regular monthly cyclic menses (cycles occurring every 25–35 days). Subjects were excluded if they had previously suffered a low trauma fracture (as judged by the investigator), any metabolic bone diseases, or chronic diseases capable to influence bone metabolism (malignancies, rheumatoid arthritis, diabetes, etc.). Subjects were also excluded if abnormal laboratory results in serum calcium, creatinine, phosphate, and magnesium were found upon screening.

The study sites received formal approval from the local Ethics Committees and obtained signed informed consent from each subject before enrolment.

### Clinical examination

The subjects considered to be eligible for inclusion in the study were asked to come to the outpatient clinic by 7:30–8:30 a.m. in fasting condition for the collection of a blood sample. Six aliquots of serum samples were separated and kept on dry ice during transportation by courier to Interlab (Munich, Germany), where they were kept at -80°C for the later measurements of bone turnover markers in all subjects, and of follicle stimulating hormone (FSH) and serum estradiol in women aged >39 yrs. It was preplanned that women with FSH levels >30 IU/L, despite menstruating normally, would be analyzed separately and defined as "perimenopausal".

After breakfast, organized locally, a Dual X-ray (DXA) evaluation was carried out and a multi-item questionnaire administrated. Hologic (20 centers) and Lunar (3 centers) instruments were used for DXA evaluation of BMD at the lumbar spine (LS), femoral neck (FN) and total hip (TH). The values obtained with Lunar instruments were standardized to Hologic instruments [10]. The questionnaire included personal data and evaluated factors that potentially influence bone turnover, including general health and any type of continuous use of drugs (including oral contraceptives or calcium supplements), fracture history, family (i.e. first degree relative) history of low-energy fractures and/or of osteoporosis (defined by LS- and/or FN-BMD<-2.0 SDS), number of pregnancies, smoking, alcohol consumption, sunlight exposure and menstrual cycle day. In all subjects body Ht and weight were assessed (Harpender stadiometer) and the body mass index (BMI, kg/m^2^) was derived.

The bone turnover markers investigated in this study were serum C-telopeptide of type I collagen (CTX), osteocalcin (OC), and N-terminal propeptide of type I procollagen (P1NP). The three bone turnover markers, FSH, and estradiol were measured by automated immunoassay with the ECLIA device from Roche Diagnostics (Palo Alto, CA, USA). The coefficients of variation (interassay) provided by Interlab ranged from 7 to 14%. Additional biochemical tests performed by local laboratories included serum calcium, creatinine, phosphate, and magnesium.

### Genotyping

Genomic DNA was extracted from peripheral blood lymphocytes using a column microvolume system (NucleoSpin Blood Quick Pure, Macherey-Nagel, Easton, PA, USA) according to the manufacturer's instructions. Genomic DNA regions of *ESR1 *and *ESR2 *genes, containing the above described polymorphisms were analyzed by polymerase chain reactions (PCR), using specific couples of primers designed by Primer3 (v.0.4.0) program, freely available [11].

#### ESR1 gene polymorpisms

Intron 1 region containing both the *rs2234693 *and *rs9340799 *polymorphisms has been amplified by PCR in a final volume of 50 μl containing 1× of reaction buffer, 0.4 μM of each primer, 0.2 mM of dNTPs, 1 U of GoTaq^® ^DNA Polymerase (Promega, Madison, WI – USA) and about 50 ng of genomic DNA. Thermal cycling conditions were 94°C for 5 min, 35 cycles of 94°C for 30 sec, 60°C for 30 sec and 72°C for 1 min, followed by an additional 72°C for 5 min stabilization step. Two aliquots of PCR products were separately digested overnight at 37°C with 1 U of *PvuII *or *XbaI *(MBI Fermentas, Vilnius, Lithuania). *PvuII *digestion products were visualised by 3% ethidium bromide stained agarose gel electrophoresis. Fragments were separated depending on their length revealing presence or absence of the restriction site and identifying respectively the *T *and the *C *alleles. Similarly, *XbaI *digestion products were visualised by 3% ethidium bromide stained agarose gel electrophoresis. Fragments were separated depending on their length revealing presence or absence of the restriction site and identifying respectively the *A *and the *G *alleles.

#### ESR2 gene polymorphism

PCR amplification was performed in a final volume of 50 μl containing 1× of reaction buffer, 0.4 μM of each primer, 0.2 mM of dNTPs, 1 U of GoTaq^® ^DNA Polymerase (Promega, Madison, WI – USA) and about 50 ng of genomic DNA. Thermal cycling conditions were 94°C for 5 min, 35 cycles of 94°C for 30 sec, 60°C for 30 sec and 72°C for 30 sec, followed by an additional 72°C for 5 min stabilization step. The 168 bp PCR product was digested over-night at 37°C with 1 U of *AluI *endonuclease (MBI Fermentas, Vilnius, Lithuania). *AluI *digestion products were visualised by 3% ethidium bromide stained agarose gel electrophoresis. Fragments were separated depending on their length revealing presence or absence of the restriction site and identifying respectively the *A *and the *G *alleles.

### Statistical analyses

Data were expressed as mean ± standard deviation (SD) unless otherwise stated. Statistical evaluation was performed using standard Chi-squared (χ^2^) test, one-way analysis of variance (ANOVA) and Pearson's correlation (*r*) where multiple samples were obtained. When two sets of data were compared, an unpaired Student's *t*-test was employed. A two-tailed significance test was used for all comparisons. Standard χ^2 ^test was also used to compare observed genotype frequencies with those expected under the Hardy-Weinberg (HW) equilibrium [12]. *P *< 0.05 was considered statistically significant. All analyses were performed using the SAS statistical package, version 8.2 (SAS Institute, Cary, NC).

## Results

A total of 641 healthy premenopausal women meeting the inclusion and exclusion criteria were recruited from 20 investigative sites. Two women were excluded due to serum calcium >10.5 mg/dL and one patient for P1NP and OC levels three times above the upper normal range, who is now under investigation for suspected Paget disease of the bone. No other patients had abnormal values of serum phosphate, magnesium or creatinine (data not shown).

Serum FSH >30 IU/L was found in 18 women even though menstruating normally. They are considered per protocol as perimenopausal. Twelve subjects were on treatment with stable doses of thyroxine, 11 on antihyperthensive agents not associated with diuretics, 3 on serotonin-uptake inhibitors, and 2 on proton pump inhibitors. Eighty-three women were on oral contraceptive treatment [9].

Genetic data were available for 573 enrolled subjects. Three non-Caucasian women were excluded from genetic analyses and their exclusion did not modify the results of the study (data not shown). Statistical analysis was performed on the remaining 570 Caucasian women who were not on treatment with known bone-active drugs.

The general characteristics of the study population on the basis of allelic variability for *ESR1 *and *ESR2 *loci are described in Table 1. According to [12], genotype distributions of these two loci were found to be in HW equilibrium (data not shown), suggesting that the enrolled subjects represented a homogeneous genetic background. No difference was evidenced in *ESR1 *and *ESR2 *loci for all considered variables, though a tendency to on increased Ht and a delayed menarche age were detected for *ESR1 rs2234693 CC*, *rs9340799 GG *or *CCGG*, and *ESR2 AA *genotypes than for the opposites (*P *> 0.05, data not shown). Moreover, the combination of *ESR1 CCGG *plus *ESR2 AA-AG *genotype was significantly taller (164.2 ± 6.06 cm) than *ESR1 TTAA *plus *ESR2 GG *genotype (161.7 ± 6.74 cm; *P *= 0.044). When *ESR1 *and *ESR2 *genotypes were evaluated alone or in combination, no significant correlation was observed with weight, BMI, heart rate and blood pressure (*P *> 0.05).

**Table 1:** Genotype variability for *ESR1 *and *ESR2 *loci in the 570 Caucasian women enrolled in the BONTURNO study.

**Locus**	**Women (*n *= 570)**
*ESR1 rs2234693*	
CC (n [%])	123 (21.6)
CT (n [%])	287 (50.3)
TT (n [%])	160 (28.1)
ESR1 rs9340799	
AA (n [%])	203 (35.6)
AG (n [%])	272 (47.7)
GG (n [%])	95 (16.7)
*ESR2 rs4986938*	
AA (n [%])	92 (16.2)
AG (n [%])	266 (46.7)
GG (n [%])	212 (16.7)

Regarding family history of osteoporosis (FHO) and of hip fracture (FHF), no significative association was found with single or combined analysis of *ESR1 *polymorphisms. In subjects positive both for FHO and for FHF, the 3 *ESR1 rs2234693 *genotypes (but not the *rs9340799*) were differently distributed than in subjects with double negative FHO-FHF (χ^2 ^= 10.957, *P *< 0.01; Table 2), with odds ratio (OR) of founding *ESR1 CT*-*TT *genotypes in double positive FHO-FHF being 1.836 (95% CI 0.860–3.919, *P *= 0.06). No significant association was detected combining both *ESR1 *polymorphisms (*P *> 0.05). Furthermore, *ESR2 rs4986938 *genotypes correlated with FHF (χ^2 ^= 11.881, *P *< 0.01) but not with FHO (*P *> 0.05), as having at least one *ESR2 rs4986938 A *allele correlated both with positive FHF (χ^2 ^= 11.550, *P *< 0.001; OR 2.387, 95% CI 1.432–3.977) and with double positive FHO-FHF (χ^2 ^= 9.407, *P *< 0.005; OR 2.871, 95% CI 8.804–35.403) (Table 2). No association was detected co-analyzing *ESR1 *and *ESR2 *genotypes (*P *> 0.05).

**Table 2:** Family history of osteoporosis (FHO) and of hip fracture (FHF) regarding *ESR1 *and *ESR2 *genotypes.

	**FHO**	**FHF**	**FHO-FHF**	**FHO**	**FHF**	**FHO-FHF**
	positive			negative		
*ESR1 rs2234693*						
CC (n [%])	39 (17.6)	18 (17.6)	9 (14.7)	77 (24.0)	98 (22.1)	68 (24.1)
CT (n [%])	118 (53.1)	50 (49.1)	32 (52.5)	158 (49.0)	227 (51.2)	141 (50.0)
TT (n [%])	65 (29.3)	34 (33.3)	20 (32.8)	87 (27.0)	118 (26.7)	73 (25.9)
Total	222	102	61	322	443	282
*ESR2 rs4986938*						
AA (n [%])	36 (16.3)	18 (18.2)	12 (19.7)	50 (15.8)	68 (15.5)	44 (15.8)
AG (n [%])	104 (47.1)	59 (59.6)	38 (62.3)	119 (37.5)	193 (44.0)	127 (45.5)
GG (n [%])	81 (36.6)	22 (22.2)	11 (18.0)	148 (46.7)	178 (40.5)	108 (38.7)
Total	221	99	61	317	439	279

The 570 enrolled women were divided in 3 age groups: from 20 to 30 yrs (class 1), from 31 to 40 yrs (class 2) and from 41 to 50 yrs (class 3) (Table 3). In class 1, subjects having at least one *ESR1 rs2234693 C *allele (*i.e. CC *and *CT *genotypes) presented lower LS-BMD (1.023 ± 0.112 g/cm^2^) and TH-BMD (0.927 ± 0.122 g/cm^2^) than *TT *genotypes, respectively (LS-BMD 1.077 ± 0.131 g/cm^2^, *P *= 0.0083; TH-BMD 0.969 ± 0.121 g/cm^2^, *P *= 0.0474). Similar but not significant trends were detected in classes 2 and 3. In class 3, TH-BMD (0.860 ± 0.111 g/cm^2^) and FN-BMD (0.738 ± 0.108 g/cm^2^) of *ESR2 rs4986938 AA *genotype were lower than the opposite *GG *ones (TH-BMD 0.923 ± 0.130 g/cm^2^, *P *= 0.0227; FN-BMD 0.798 ± 0.119 g/cm^2^, *P *= 0.0180). Regarding LS-, TH- or FN-BMD, no other signifivative differences were observed co-analyzing *ESR1 *and *ESR2 *loci.

**Table 3:** Mean age-class adjusted values (SD) of LS-, TH- and FN-BMD regarding *ESR1 *and *ESR2 *genotypes.

Age classes	1			2			3		
Subjects (n)	158			192			219		
Age range (yrs)	20–30			31–40			41–50		
BMD (g/cm2)	**LS**	**TH**	**FN**	**LS**	**TH**	**FN**	**LS**	**TH**	**FN**
*ESR1 rs2234693*									
CC	1.063 (0.134)	0.941 (0.117)	0.859 (0.150)	1.075 (0.138)	0.906 (0.098)	0.821 (0.125)	1.021 (0.122)	0.902 (0.115)	0.778 (0.122)
CT	1.025 (0.112)	0.930 (0.120)	0.845 (0.116)	1.067 (0.122)	0.906 (0.117)	0.795 (0.118)	1.041 (0.127)	0.905 (0.127)	0.789 (0.115)
TT	1.077 (0.131)	0.969 (0.121)	0.874 (0.129)	1.059 (0.106)	0.928 (0.105)	0.813 (0.118)	1.049 (0.125)	0.904 (0.124)	0.777 (0.122)
*ESR2 rs4986938*									
AA	1.039 (0.130)	0.932 (0.110)	0.846 (0.112)	1.059 (0.123)	0.922 (0.110)	0.814 (0.122)	1.044 (0.147)	0.860 (0.111)	0.738 (0.108)
AG	1.044 (0.126)	0.935 (0.125)	0.847 (0.131)	1.076 (0.117)	0.910 (0.106)	0.804 (0.110)	1.032 (0.128)	0.900 (0.118)	0.784 (0.118)
GG	1.056 (0.121)	0.963 (0.122)	0.870 (0.135)	1.056 (0.125)	0.911 (0.115)	0.806 (0.132)	1.046 (0.115)	0.923 (0.130)	0.798 (0.119)

According to previously published data, oral contraceptive users and 18 women considered in perimenopausal phase for serum FSH levels >30 IU/mL were excluded from statistic analysis for bone turnover markers [9]. Age class-adjusted levels of serum OC, CTX and P1NP did not segregate with *ESR1 *and *ESR2 *loci (*P *> 0.05). Furthermore, no differences between *ESR1 *and *ESR2 *polymorphisms, were detected for serum age class-adjusted levels of calcium, phosphate and magnesium (*P *> 0.05).

## Discussion

Low BMD is a major risk factor for spine and proximal femur fractures [13,14]. In women, BMD in adulthood is largely determined by the amount of bone accumulated at the end of their skeletal growth (peak bone mass), their rate of bone loss after menopause when ovaries cease producing estrogens, and age-related bone loss. It has been well established with the study of twins, that peak bone mass is highly heritable with an estimated heritability between 0.50 and 0.80 [15]. Conversely, published data on the heritability of bone loss at menopause are conflicting [16-18]. Therefore, BMD is a trait that lends itself to studies designed to identify the genes underlying its normal variation [16,17,19].

The past decade has seen an important increase in the use of association studies with candidate genes for the genetic analysis of complex traits such as BMD and/or fracture risk. Many genes have been examined for their association with normal BMD variation, which yields an ever-expanding candidate gene list. However, this approach has been largely criticized because of discrepancy in the results [20,21], often related to the small size of the enrolled cohorts. Moreover, most of the studies focused on postmenopausal female populations. In this view, the main purpose of the present study was to evaluate allelic influence of target genes, such as estrogen receptors, on inherited skeletal traits in a large and homogeneous population-based cohort of premenopausal healthy Caucasian women [9].

For *ESR1 *and *ESR2*, two genes worldwide evaluated by independent research groups, the results obtained even if compelling for their involvement in BMD, osteoporosis, or fracture, are, however, not conclusive [22]. Confounding factors encompassed ethnic-specific distribution of *ESR1 *and *ESR2 *polymorphisms [8,23,24]. For example, in the SWAN study [23] which enrolled 693 Caucasian participants (366 premenopausal women), specific associations of BMD with *ESR1 *and *ESR2 *genotypes varied according to race/ethnicity. Furthermore, 4 independent studies [24] concluded that *ESR2 *locus could be involved in FN-BMD in Caucasians, LS-BMD in Japanese postmenopausal women, and LS- and FN-BMD in Chinese premenopausal women. In addition, most of the human studies of genetic association with BMD have been cross-sectional, and only very few studies examined the association of the genotypes to BMD change within specific age ranges. For all these reasons the present study was aiming to compare the results obtained for *ESR1 *and *ESR2 *to other data obtained in age-equivalent studies in Caucasian women, examining the relation of *ESR1 *and *ESR2 *genes' polymorphisms.

Differently than for the *ESR2 *locus [25], allelic variants of *ESR1 *gene were proposed to affect skeletal growth, through a genotype-dependent estrogen sensitivity at the growth cartilage, with the *ESR1 px *haplotype being less sensitive to estrogen effects [26]. The *ESR1 *haplotype effect was supported by functional studies [27,28] and by multiple association analysis documented for this gene variant [29-33]. For example, body Ht in pre- and postmenopausal women [29] and estradiol levels in premenopausal women were lower [30], with the number of copies of *ESR1 px *haplotype in their genotype. Lorentzon et al. [31] found an association between reduced Ht and *Pvu*II *T *and *Xba*I *A *alleles, which corresponded to the *ESR1 px *haplotype. Although this study was performed in adolescent boys [31], it is in line with other findings in adult women. In 607 Caucasian women (aged 55–80 yrs) in whom vertebral fractures were excluded, Schuit et al. [29] observed significant association between Ht and *ESR1 Pvu*II-*Xba*I haplotypes. In contrast to [32], a significant allele dose effect was observed for *ESR1 px *haplotype, corresponding to a 0.9-cm decrease in Ht per allele copy (*P *for trend = 0.02), extreme genotypes varied 1.8 cm. Boot et al. [33] partially confirmed this allele dose-effect to some extent, as in girls heterozygous for *ESR1 px *haplotype the Ht was higher than in those homozygous for the *ESR1 px *haplotype. In our series, higher Ht was slightly (*P *> 0.05) correlated with *ESR1 CC*, *GG *or *CCGG*, and *ESR2 AA *genotypes, while *ESR1 CCGG *plus *ESR2 AA-AG *genotype was significantly (2.5-cm) taller than the opposite genotype. As *ESR2 *modulates *ESR1 *transcriptional activity [34], this novel biological interaction between *ESR2 *and *ESR1 *genotypes is not surprising.

Family history is a major risk factor for osteoporotic fractures [35]. In white postmenopausal women, increased BMD-independent risk for vertebral (but not non-vertebral) fractures was found in *ESR1 px *haplotype carriers [26]. Moreover, the GENOMOS Consortium found a BMD-independent protective effect against vertebral fractures in *ESR1 XX *homozygous individuals, while no effects on fracture risk were seen for *ESR1 Pvu*II polymorphism [22]. Similarly to the InCHIANTI study [36], we could not demonstrate any strong association between FHF and *ESR1 rs2234693 *and *rs9340799 *genotypes. However, our study might have had not enough power to detect any differences.

Variants of *ESR2 *gene, alone and in interaction with *ESR1 *genotypes influenced the fracture risk in postmenopausal women. Moron et al. [37] suggested that *ESR2 rs4986938 *(but not *ESR1 rs2234693*) could have a role (*P *= 0.04) in osteoporosis in Spanish postmenopausal women. Furthermore, they detected a joint effect of *ESR1 *gene in osteoporosis modulating the penetrance of *ESR2 rs4986938 *genotype [37]. Rivadeneira et al. [38] showed for the first time that white postmenopausal women (≥ 55 yrs of age) who are homozygous for a common intron 2–3'*UTR ESR2 *haplotype allele have 40–80% increased risk of fragility and vertebral fracture. Interestingly, we also observed *ESR2 rs4986938 *genotypes significantly correlated with FHF risk but not with FHO, suggesting *ESR2 *variants may affect bone strength independently of BMD.

According to our findings, McGuigan et al. [39] observed a modest association between *ESR1 Pvu*II genotypes and BMD at the hip (*P *= 0.034) but not at the spine in 216 young Irish women (mean age 22.6 ± 1.6 yrs), with no differences regarding the *ESR1 Xba*I locus [39]. On the other hand, Valero et al. [40] found no significant relations between FN- or LS-BMD with both *ESR1 Pvu*II and *Xba*I loci in 194 older Caucasian women aged 22–45 yrs. Furthermore, a cross sectional study of *Xba*I and BMD in women who were premenopausal and perimenopausal, did not confirm this association [41]. Finally, in perimenopausal Caucasian women (older than 48.5 yrs) enrolled in the GENOMOS consortium, none of two *ESR1 *intron 1 polymorphisms (*i.e. Pvu*II and *Xba*I loci) or derived haplotypes had any statistically significant effect on BMD, with estimated differences between genetic contrasts being 0.01 g/cm^2 ^or less [22]. Collectively, our findings and the published studies [22,39-41] make possible to support a significant effects of the *ESR1 rs2234693 *(but not *rs9340799*) locus on the BMD mainly in the young adult next to her achievement of bone peak mass.

Previous approaches have also suggested the role of *ESR2 *in BMD within different ethnic backgrounds [24]. No association between *ESR2 rs4986938 *with LS- or FN-BMD were detected in 1291 Caucasian women (from 192 families) aged 33.2 ± 7.1 yrs (range 20–50 yrs) [42]. Similarly, no associations between *ESR2 rs4986938 *genotypes and Ht, LS-BMD and serum OC levels were detected in 147 healthy peri and postmenopausal Greek women (mean age 54 ± 7.9 yrs) [25]. On the other hand, we detected significant BMD variations of the *ESR2 rs4986938 *genotypes only in the later age group (i.e. 41–50 yrs old women). Together with *ESR1 rs2234693 *data, this reinforces the hypothesis that *ESR1 *and *ESR2 *genes affect bone metabolism in precise and distinct age-sequential windows. Larger pre-planned analysis will be necessary to confirm our interpretation.

In conclusion, taken together, our findings indicated that, although the effect size may be small, allelic variations in *ESR1 *and *ESR2 *genes are associated with various and different bone traits (e.g. Ht, BMD and FHF risk) in normal premenopausal Caucasian subject. Furthermore, multiple genotype interactions were detected that reinforced the polygenic and complex character of skeletal system. In some cases however, the mean pattern of bone trait values for a gene polymorphism with evidence of association was not in agreement with previously published studies. Therefore, even though family history of fragility fractures is one of the risk factors [35], we cannot recommend genetic testing for clinical use in humans to better identify population at risk for pathologic bone traits such as fragility fractures. However, as it has been shown for other diseases [43], extended panels of several polymorphic markers could be used in the future, in addition to traditional risk factors, to evaluate the skeletal disorder risk in humans.

## Competing interests

All authors participating to the BONTURNO study, and then to the preparation of this manuscript, did not have competing interests regarding the present data.

## Authors' contributions

Both FM carried out the molecular genetic studies, performed the statistical analysis and drafted the manuscript. LM participated in the sequence alignment. GB, SM, GL and LM carried out the subject enrolment and their clinical evaluation. AP, SS and DM participated in the design of the study and in the subject data collection. MLB conceived of the study, and participated in its design and coordination and helped to draft the manuscript. All authors read and approved the final manuscript.
